# PDGFRβ Expression Across Canine AGASAC Subtypes and Metastases: Morphologic Insights and Possible Therapeutic Implications †

**DOI:** 10.3390/vetsci12121122

**Published:** 2025-11-26

**Authors:** Silvia Dell’Aere, Alessandra Verdi, Clarissa Zamboni, Damiano Stefanello, Roberta Ferrari, Elisa Maria Gariboldi, Lorella Maniscalco, Selina Iussich, Caterina Romanello, Valeria Grieco, Chiara Giudice, Camilla Recordati, Andrea Cappelleri, Valeria Buonfrate, Paola Roccabianca

**Affiliations:** 1 Department of Veterinary Medicine and Animal Sciences—DIVAS, University of Milan, 26900 Lodi, Italy; silvia.dellaere@unimi.it (S.D.); alessandra.verdi@unimi.it (A.V.); damiano.stefanello@unimi.it (D.S.); elisamaria.gariboldi@unimi.it (E.M.G.); valeria.grieco@unimi.it (V.G.); camilla.recordati@unimi.it (C.R.); andrea.cappelleri@unimi.it (A.C.); 2 Department of Veterinary Sciences, University of Turin, 10095 Grugliasco, Italy; lorella.maniscalco@unito.it (L.M.);; 3 Department of Veterinary Medicine, University of Bari, 70010 Valenzano, Italy

**Keywords:** anal sac apocrine gland adenocarcinoma, dog, molecular targets, platelet-derived growth factor receptor beta, Receptor tyrosine kinases, toceranib phosphate

## Abstract

Canine apocrine gland anal sac adenocarcinoma (AGASAC) is an aggressive malignancy with a high incidence of regional lymph node metastasis at diagnosis. Platelet-derived growth factor receptor beta (PDGFRβ) is a receptor tyrosine kinase involved in oncogenic signaling and angiogenesis, representing a potential therapeutic target. Its expression in different AGASAC histotypes has not been fully defined. This study evaluated microscopic patterns and PDGFRβ immunohistochemical expression in three normal anal gland sacs, 51 primary AGASACs (12 non-metastatic, 39 metastatic), and 33 corresponding nodal metastases. PDGFRβ expression was semi-quantitatively scored and statistically analyzed. Histotypes included 26 mixed with solid prevalence, 11 pure solid (including 1 comedo-carcinoma), 9 mixed with tubular prevalence, 4 tubular, and 1 neuroendocrine-like. PDGFRβ was expressed in normal anal sac glands and in 43/51 tumors (5–100% positive cells): 19/26 mixed with solid prevalence, 10/11 solid, 9/9 mixed with tubular prevalence, 4/4 tubular, and 1 neuroendocrine-like. PDGFRβ labeled 27/33 nodal metastases. PDGFRβ expression was highest and more intense in tumors with a tubular pattern. The statistical trend indicated a correlation of AGASAC dedifferentiation to reduced PDGFRβ expression, but no statistical significance was found. Rare AGASAC variants (solid comedo-carcinoma and neuroendocrine-like) were described for the first time. PDGFRβ expression was higher in tubular tumors and commonly detected in primary and metastatic lesions. These findings support exploring TKI therapy for specific AGASAC histotypes. Further studies integrating receptor activation and treatment outcomes are warranted to clarify predictive and prognostic relevance.

## Simple Summary

Dogs can develop a tumor called apocrine gland anal sac adenocarcinoma, which often spreads to lymph nodes and other body parts. In a study of 51 dogs, we examined the protein PDGFRβ on tumor cells. This protein promotes tumor growth and new blood vessel formation. We found that about 84% of primary tumors expressed PDGFRβ, both in the cancer cells and surrounding supportive tissues like blood vessels. When the cancer metastasized, it usually formed more solid tumors with the same or lower levels of PDGFRβ compared to the original tumor. These findings are significant because existing drugs called tyrosine kinase inhibitors target PDGFRβ. The results suggest these drugs could benefit some dogs with this cancer. However, tumors that become more solid and lose PDGFRβ may respond less effectively to treatment. Future studies linking PDGFRβ levels to treatment outcomes could help identify which dogs are most likely to benefit from targeted therapy.

## 1. Introduction

AGASAC is a malignant neoplasia characterized by frequent regional lymph node metastasis (72–94% of cases) [1,2], distant metastasis (21–45% of cases) [3,4,5], especially to the spleen, liver, and lungs [1,6,7], and hypercalcemia (25–53% of cases) [1,7,8,9] at diagnosis in affected dogs [8].

Despite the use of multimodal therapeutic approaches—including surgery, radiation therapy, and systemic chemotherapy—disease progression with relapses and nodal and distant metastasis is frequent. Tumor-related death is common, and in cases with lymph node metastases at diagnosis, the reported mean survival time does not exceed a year [7]. This underscores the need for novel therapeutic approaches to control nodal and distant disease using additional strategies such as those targeting molecular pathways implicated in tumor growth and progression.

Receptor tyrosine kinases (RTKs) are a family of transmembrane receptors, including vascular endothelial growth factor (VEGF) receptor (VEGFR), platelet-derived growth factor receptors (PDGFRs), and KIT, that act as critical regulators of cellular processes such as proliferation, differentiation, and survival, and their dysregulation is a hallmark of many cancers [10,11]. Among the RTKs, PDGFRs play a role in tumoral angiogenesis by stimulating VEGF expression in tumor-associated blood vessel endothelium and by recruiting pericytes [12]. The aberrant activation of PDGFR is linked to tumorigenesis and metastatic progression in malignancies such as gliomas, sarcomas, and gastrointestinal stromal tumors [13,14]. In veterinary medicine, the role of PDGFR, particularly PDGFRβ, is gaining recognition as a therapeutic target, evidenced by its expression in canine malignant tumors such as hemangiosarcomas [15], AGASACs and thyroid carcinomas [16], and urinary bladder transitional cell carcinomas [17]. AGASACs were reported as prevalently PDGFRβ-negative, with only one case reported as intensely positive [16,18].

The therapeutic relevance of PDGFRβ in canine cancers is further supported by the efficacy of treatment with RTK inhibitors (TKIs) such as toceranib phosphate, which targets PDGFRβ among other kinases, for various canine malignancies [19], including mast cell tumors [20], osteosarcoma, and thyroid carcinoma [20]. Acting as a competitive inhibitor of ATP, toceranib phosphate prevents the RTK receptor phosphorylation and the downstream transduction of the signal. The empirical clinical use of TKIs in the therapy of AGASACs and preliminary clinical studies have reported the efficacy of toceranib phosphate alone, or as adjunct therapy in AGASACs, with clinical improvements or increased survival time [20,21,22,23].

However, only a limited number of studies have investigated the expression and functional role of TKIs’ molecular targets, such as PDGFRβ [16,18,23], in AGASAC and corresponding lymph node metastasis [20,22], and to our knowledge PDGFRβ has never been investigated in relation to the histopathological growth patterns of this malignancy.

Given the aggressive nature of AGASAC and the limited success of conventional therapies, understanding the role of PDGFRβ in AGASAC could provide additional support for the use of RTK inhibitors in this tumor type.

This study aims to assess PDGFRβ expression in canine primary AGASACs and corresponding lymph node metastases, in relation with the histological growth pattern, to explore the potential relevance of PDGFRβ as a therapeutic target. The secondary aim of this study is to investigate and provide evidence supporting the development of PDGFRβ-targeted therapies to improve the prognosis of dogs diagnosed with AGASAC.

## 2. Material and Methods

### 2.1. Case Selection

Samples of canine primary AGASAC at first presentation and their corresponding regional lymph node (when available) were retrospectively selected between 2011 and 2024 from the archives of the Department of Veterinary Medicine and Animal Sciences (DIVAS) of the University of Milano (Italy) and from the archives of the Department of Veterinary Sciences of the University of Torino (Italy). Keywords for the electronic database search included dog, apocrine, and anal sac.

For each dog, data regarding signalment, clinical presentation, and tumor site were collected. Information regarding lymph node enlargement and the presence of lesions suggestive of distant metastatic sites was collected by whole-body CT scans. Regarding normal-sized vs. enlarged lymph nodes, they were defined according to Palladino et al. (2016) (enlarged) [24] and Teodori et al. (2021) (normal-sized) [25].

Non-neoplastic anal sacs obtained from necropsies were also selected from the archives of the Department of Veterinary Medicine and Animal Sciences (DIVAS) of the University of Milano (Italy).

### 2.2. Histopathology

All samples submitted for histological evaluation were reviewed by 4 pathologists (of which 1 was board-certified [PR], 2 were ECVP residents [SD, AV], and 1 was a DVM [LM]) examining H&E-stained 4 μm sections of formalin-fixed paraffin-embedded (FFPE) routinely processed tissues to confirm the diagnosis of AGASAC. 

Complete excisional samples of primary AGASAC and, when excised, the corresponding regional lymph nodes were received, and a cross section of the primary tumor or of the metastatic lymph node was examined across the entire available area (for pattern assessment) at 20× magnification by systematically scanning the sections utilizing always the same 10-headed microscope (Olympus BX51) with ocular magnification of 10×/22.

Growth patterns were assessed by scanning the entire available surface of the routinely stained sections at 20× magnification, and the prevalence of tumor growth pattern was recorded as pure solid with a subtype of pure solid with central necrosis (comedo-carcinoma), pure tubular, mixed with solid prevalence, mixed with tubular prevalence, and pure neuroendocrine-like (rosettes and packets, this last prevailing) [5]. Necrosis was assessed as present or absent. For cases where discrepancies arose among individual observers, a re-evaluation was performed at the 10-headed microscope (Olympus BX51) at 20× magnification (ocular magnification of 10×/22) by 3 pathologists (SD, AV, PR) to reach a consensus.

### 2.3. Immunohistochemistry

PDGFRβ expression was evaluated on 4 μm paraffin sections of the primary AGASAC and metastases, mounted on poly-L-lysine-coated slides [26,27], and stained with rabbit polyclonal anti-human PDGFRβ primary antibody (3162S, Cell Signaling Technology, Danvers, MA, USA). Cross-reactivity with canine tissues was explored by aligning human and canine sequences, demonstrating sequence identity of 89.7552% (CLUSTALW alignment, Supplemental Material S3). Cross-reactivity of the markers has been validated by Western blotting [28] and utilized for other canine tumor types in vivo and in vitro [28,29].

Briefly, sections underwent rehydration and deparaffinization through an ascending alcohol series, followed by quenching of endogenous peroxidase with 3% hydrogen peroxide and antigen retrieval in a preheated Tris-EDTA buffer (pH 9) at 97 °C for 25 min. Sections were incubated with anti-PDGFRβ primary antibody at 1:100 dilution for 1 h, then with biotinylated secondary antibody (goat anti-rabbit, BA1000, Vector, CA, USA) at 1:200 dilution for 30 min, followed by avidin–biotin enzyme complex (ABC kit, PK6100, Vectastain, Vector, CA, USA) incubation according to the manufacturer’s instructions for 30 min. All the incubation steps were performed at room temperature. The reaction was visualized with the diaminobenzidine substrate kit (ImmPACT DAB kit, SK4105, Vector, CA, USA) as per the manufacturer’s instructions. Sections were counterstained with Mayer’s hematoxylin (Diapath, C0302, Bergamo, Italy), dehydrated, and mounted with Micromount (Diapath, 060500, Bergamo, Italy). A PDGFRβ-positive canine hemangiosarcoma was included in every run as the positive control. Negative controls consisted of primary antibody replacement with an isotype-matched irrelevant antibody (rabbit anti-Von Willebrand Factor, ab6994, AbCam, Cambridge, UK).

The staining was semi-quantitatively evaluated, visually estimating the percentage of immunolabeled neoplastic cells (0, negative <5%; 1, 5–25%; 2, 26–50%; 3, 51–75%; 4, >75%) [30,31], and cancer-associated stromal microenvironment, including cancer-associated fibroblasts (CAFs), blood vessels (negative, <5% of positive cells; low positive, 6–50%; strongly positive, >50%), and stroma [32,33,34] for all cases by utilizing previously reported methods [30,31,32,33,34].

Immunohistochemical evaluation included a semi-quantitative visual estimation of staining intensity (weak, intermediate, strong), following previously described methods [35,36]. When mixed intensities were present, cases were assigned to the higher category. Intensity scoring was performed using the strongest-staining cases and the negative controls as references, with each case evaluated at least twice and a final, simultaneous third review conducted in the same session for all cases.

Immunohistochemical evaluation and score attribution were independently performed by 3 pathologists (SD, AV, PR). For cases with mixed stain intensity, the intensity was assigned to the higher class. For cases with scoring discrepancies, a joint re-evaluation was performed at the 10-headed microscope (Olympus BX51) at 20× magnification (ocular magnification of 10×/22) by the same pathologists to reach a consensus.

### 2.4. Statistical Analysis

Statistical analysis was conducted using JASP statistical software (ASP Team. JASP (version 0.19.3) [computer software]. Multiclass analysis and descriptive statistics were calculated for PDGFRβ expression scores for all tumor histotypes. Given the violation of normality assumptions (Shapiro–Wilk tests *p* < 0.001), the Mann–Whitney U test was performed with the level of significance of *p* < 0.05.

### 2.5. Use of GenAI

ChatGPT (model GPT-5) was utilized to correct grammar, spelling, and syntax prior to submission of the manuscript. Additionally, ChatGPT was utilized to refine the graphical abstract: specifically, the drawing of the dog neoplastic mass, of the lymph node metastases, and of the microscope and antibody.

## 3. Results

### 3.1. Cases

A total of 51 AGASACs, from 51 dogs, were included in this study. Of these, 39/51 (76.5%) were associated with lymph node enlargement assessed by whole-body computed tomography (CT). Samples from all primary masses and from 33 of the enlarged lymph nodes were available for histological assessment and were included in this study.

Signalment and clinical presentation are listed in Table 1 and detailed in Supplemental Table S1.

Briefly, most dogs were older females; breeds comprised twenty-two mixed breeds (43%), nine Labrador Retrievers (18%), four Border Collies (8%), and Czechoslovakian Wolfdogs, Cavalier King Charles Spaniels, and German Shepherds (two of each, 4%). The remaining breeds were represented by one dog each (2%): German Dachshund, Basset Hound, Beagle, Boxer, Cocker Spaniel, Golden Retriever, Jack Russel Terrier, English Setter, Siberian Husky, and Hungarian Vizsla.

A higher number of tumors involved the right anal sac, and lymph node metastases were frequent. At excision, the size of the primary mass ranged from 0.5 cm to 11 cm, with a mean of 4.07 cm.

At presentation, whole-body computed tomography (CT) showed enlarged regional lymph nodes in 39/51 cases (76%) and normal-sized lymph nodes in 12/51 cases (24%).

By whole-body CT scans performed at presentation, lesions compatible with distant metastases were detected in 11/51 cases (22%). In one site for six cases or in multiple sites for five dogs. Organs with lesions suspected of metastases included the spleen, lungs, ilium, liver, and vertebrae. No cytological or histological biopsies were taken to confirm the diagnosis of distant metastasis.

### 3.2. Histology

Histological findings are detailed in Table 2.

The most frequent growth pattern was the mixed growth type with a predominance of solid areas. Less represented growth patterns included purely tubular (four cases) and neuroendocrine-like growth (one case).

Necrosis was observed in 17/51 cases, scoring always less than 50%.

Of the 33 metastatic lymph nodes examined histologically, the microscopic growth pattern matched that of the corresponding primary tumor in 27 cases. In six lymph nodes the growth pattern had an increased solid component (Supplemental Table S2).

### 3.3. Immunohistochemical Expression of PDGFRβ

Immunohistochemical results of PDGFRβ expression are detailed in Table 3 for primary AGASAC and Table 4 for lymph node metastases.

**Figure 1:**
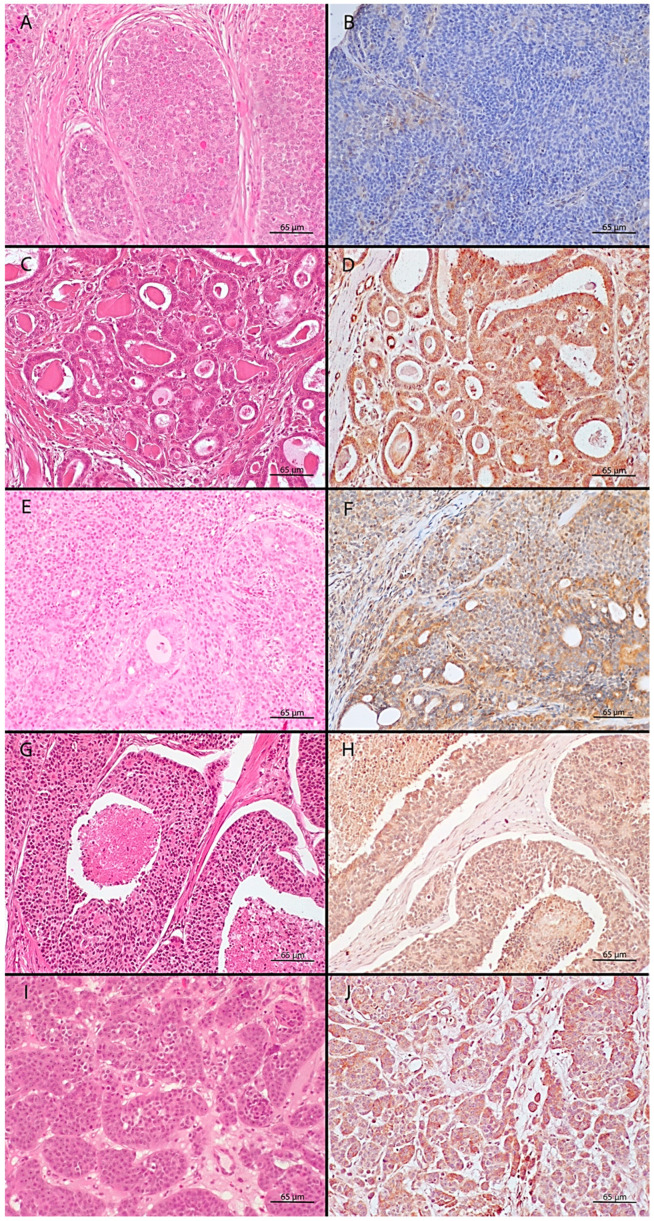
Microscopic images of canine AGASAC growth patterns. (**A**,**B**) Pure solid pattern: (**A**) Lobules of neoplastic cells with scant stroma, surrounded by desmoplasia; (**B**) PDGFRβ is variably expressed in neoplastic cells’ cytoplasm and membrane. Blood vessel endothelial cells and stromal CAFs are PDGFRβ-positive. (**C**,**D**) Pure tubular pattern: (**C**) Neoplastic cells in well-formed tubules; (**D**) PDGFRβ is strongly expressed in the tubular epithelium’s cytoplasm and endothelial cells. (**E**,**F**) Mixed pattern: (**E**) Areas with tubular and solid growth; (**F**) PDGFRβ expression is heterogeneous, with strong labeling in tubules and weaker in solid areas. CAFs are visible in the stroma. (**G**,**H**) Comedo-carcinoma pattern: (**G**) Solid sheets with necrosis; (**H**) neoplastic cells and CAFs have moderate cytoplasmic PDGFRβ. (**I**,**J**) Neuroendocrine-like pattern (rosettes and packets): (**I**) Small, scattered to confluent packets; (**J**) PDGFRβ varies, often labeling scattered cells from strong to weak. (**A**,**C**,**E**,**G**,**I**)—H&E stain; (**B**,**D**,**F**,**H**,**J**)—PDGFRβ expression, evaluated by immunohistochemistry with DAB chromogen.

Representative images of histological growth patterns and PDGFRβ expression in AGASACs are depicted in Figure 1.

**Figure 2:**
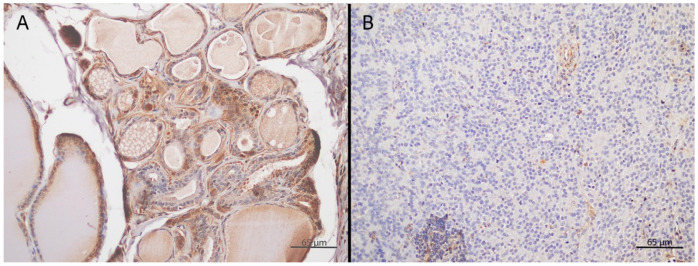
PDGFRβ immunohistochemical expression in primary AGASACs and corresponding metastasis. (**A**) Primary AGASAC with tubular growth pattern demonstrating diffuse, intense, cytoplasmic PDGFRβ expression. (**B**) Corresponding metastatic lesion in the sacral lymph node, characterized by a shift toward a more solid growth pattern and reduced, faint PDGFRβ staining.

Figure 2 illustrates PDGFRβ expression in primary AGASAC and its corresponding metastasis.

In the normal anal sacs, PDGFRβ was intensely expressed in ≥75% of the apocrine gland epithelial cells.

PDGFRβ was intensely expressed by most epithelial cells lining normal anal glands. Cells, vessels, and collagen of normal anal sac stroma were negative.

PDGFRβ was expressed in 43 primary AGASACs. Eight tumors were PDGFRβ-negative (seven were of mixed with solid prevalence, and one was purely solid).

No predominance of stain intensity was observed in relation to tumor growth pattern.

The intensity of PDGFRβ expression in normal anal sac glandular epithelium was similar to that observed in well-differentiated tubular tumors.

PDGFRβ was expressed in 27 lymph nodal metastases, while 6 were negative. Metastases generally showed a lower percentage and intensity of PDGFRβ expression than primaries. Of the positive lymph node metastases, 12 displayed similar PDGFRβ expression percentages to the primary mass. In 12 metastatic lymph nodes, PDGFRβ expression was decreased; in three metastases, PDGFRβ expression was increased compared to the primary tumors. Of the PDGFRβ-negative metastases, three were from PDGFRβ-negative primary AGASACs and three from PDGFRβ-positive AGASACs.

Only one case of tubular AGASAC with nodal metastasis was evaluated, and in this case, the metastatic cells showed reduced expression compared to both the normal anal sac epithelium and the corresponding primary tumor.

Expression of PDGFRβ in tumor-associated compartments is detailed for primary AGASAC in Table 5 and for metastatic lymph nodes in Table 6.

Cancer-associated fibroblasts and blood vessels were frequently strongly positive. Stromal extracellular matrix was consistently negative in primary tumors and metastases.

### 3.4. Statistical Analysis

Statistical analysis results are listed in Table 7 and Table 8. PDGFRβ expression scores showed variable distributions across tumor histotypes. Pure tubular and mixed with tubular prevalence cases displayed the highest median positivity (3.0–3.5) and mean ranks (31.12), indicating a trend toward stronger PDGFRβ expression. Conversely, tumors with pure solid and mixed with solid prevalence exhibited lower median scores (2.0–2.5) and mean ranks (24.25), reflecting weaker staining intensity. Although differences between groups did not reach statistical significance (Mann–Whitney U test, *p* = 0.142 and *p* = 0.121), the overall distribution pattern suggested a possible relationship between histological differentiation and PDGFRβ expression intensity.

## 4. Discussion

In this study, histopathological growth patterns and corresponding PDGFRβ expression were assessed in primary canine AGASAC and corresponding regional lymph node metastases to provide the basis for future adjunctive chemotherapeutic treatment of this malignancy with tyrosine kinase inhibitors (TKIs).

Although numerous studies have examined the histology of primary AGASAC [7], far fewer have addressed the histologic growth patterns in lymph node metastases. Existing reports only note that metastatic lesions may exhibit either a solid or a tubular or rosette architecture [2]. 

In our caseload, AGASAC had different growth patterns, with 69% mixed tumors paralleling the report of mixed growth patterns in 40–98% of cases [5,37]; in our caseload, mixed tumors with solid prevalence were more common (51%). Although a purely solid growth was not frequent (19.5%), areas of solid tumor growth were observed in 90% of the AGASACs examined, exceeding what is reported in the literature [5,7,37]. However, all studies concur in considering solid the most frequent AGASAC tumor growth [5,7]. Recognition of solid growth is clinically relevant, as it is indicative of reduced differentiation and has been linked to worse outcomes [5,37].

Rare histological patterns reported in this study included a pure solid subtype with extensive central necrosis (comedo-carcinoma) and one with purely neuroendocrine growth (packets and rosettes, the latter prevailing). To the authors’ knowledge, these growth patterns are rare and seem to have been described in this case series for the first time. Unfortunately, no conclusions regarding their biological relevance can be drawn, given their paucity.

Lymph node metastases were compared to their corresponding primary tumor, with most bearing the same growth, while 18% had increased solid growth, indicating reduced differentiation. As solid growth is reputed to be the least differentiated pattern and is associated with increased aggressiveness and worse prognosis [5,37], it is plausible that solid growth may be maintained in metastatic disease or may increase, reflecting progressive dedifferentiation and increased aggressiveness and invasive capacity. It would be interesting to review a larger multi-institutional set of AGASACs and correlate the percentage of solid areas with clinical stage before treatment and prognosis.

Three normal anal sacs demonstrated expression of PDGFRβ in most epithelial cells of normal glands with high intensity, with stroma being negative, a result that contrasts with previous published immunohistochemical studies reporting the overall negativity [16,18]. Yet, Urie et al. (2012) [16] have demonstrated PDGFRα and PDGFRβ RNA transcripts and PDGFRα protein expression in normal anal sacs [16]; thus, our finding should not be considered surprising.

PDGFRβ was expressed in 84% of primary AGASACs, and of these, 49% expressed PDGFRβ in more than 50% of the neoplastic cells, with distribution varying depending on the growth patterns. These results contrast with previous studies reporting less than 20% of AGASACs expressing PDGFRβ [18], with only one case expressing the protein in more than 50% of neoplastic cells [18]. Similarly, in another study, PDGFRβ negativity was reported in 83% of AGASACs, with no cases with more than 50% of positive neoplastic cells [16]. These contrasting results could be explained by a difference in the staining techniques and the primary antibody used. In our work, we observed a decreased staining distribution and intensity in the more dedifferentiated cells of the solid growth pattern, a newly reported finding that we could not compare with other published results. It is possible that the prevalence of solid tumor cases in the aforementioned caseloads could explain the contrasting expression data.

Among the 33 metastatic regional lymph nodes examined, nearly half (45.5%) showed PDGFRβ expression in a similar percentage of positive cells as the primary tumor, while another 45.5% had a reduced proportion of positive cells. Unexpectedly, PDGFRβ expression was increased in three metastatic lymph nodes. Noteworthy, lymph node metastases of PDGFRβ-negative primary tumors were consistently negative as well. This finding parallels a previous study by Urie et al. (2012) [16] that reported PDGFRβ expression in 1/10 (10%) of lymph node metastatic lesions, a percentage lower than that observed in primary tumors (4/24, 17%) [16]. A similar decrease in metastasis expression, compared to the corresponding primary AGASAC, has been observed for vascular endothelial growth factor 2 (VEGFR2), as 79% of the primary tumors labeled for VEGFR2 but only 60% of the corresponding lymph node metastases expressed the same receptor [16].

It is possible that, as the tumor undergoes dedifferentiation, the solid growth increases and develops metastatic behavior; thus, cells lose receptors that are normally expressed by apocrine cells of the anal sac, even if the primary tumor and its metastasis continue to express PDGFRβ RNA [16]. This mechanism might be comparable to the one reported for the epithelial component of nephroblastoma, where PDGFRα expression by epithelial cells is correlated with a better prognosis [38].

In our caseload, PDGFRβ expression was observed in CAFs and cancer-associated blood vessels of most primary tumors and lymph node metastases, but not in the stroma. Contrarily, stroma has been reported to express PDGFRβ [16,18]; unfortunately, the term “stromal positivity” has been used to refer to all compartments, including CAFs, vessels, and extracellular matrix, thus hampering true parallels.

The statistical analysis did not reveal significant differences in PDGFRβ expression among tumor histotypes, likely due to the limited number of cases per group. Nevertheless, the consistent trend toward higher expression in tumors with tubular and mixed with tubular prevalence patterns, compared to pure solid and mixed with solid prevalence histotypes, may have biological relevance. Tubular arrangement reflects a more differentiated phenotype, and the observed association could indicate that PDGFRβ expression decreases with dedifferentiation and progression toward a solid pattern. This interpretation aligns with the immunohistochemical findings in the broader dataset, where solid tumors and their metastases frequently exhibited reduced PDGFRβ staining intensity.

The moderate effect size (|r| ≈ 0.27) observed in both Mann–Whitney analyses supports the presence of a biological signal that might not reach significance, likely due to sample size limitations. Future studies involving larger, multi-institutional cohorts are warranted to confirm whether PDGFRβ expression correlates with growth pattern or tumor differentiation in AGASACs. Such data would also help clarify whether PDGFRβ could serve as a predictive marker for response to tyrosine kinase inhibitors, particularly in tumors retaining tubular features.

Efficacy of tyrosine kinase inhibitors (TKIs) in clinical practice is related to partial tumor response with improvement in signs and achievement of stable disease [21,22,23]; However, to the authors’ knowledge, the association between the use of TKIs and the expression of PDGFRβ has been only marginally investigated in canine AGASACs and its lymph node metastases [16,18].

In this study, PDGFRβ expression was evaluated in different histotypes to support the selective use of tyrosine kinase inhibitors (TKIs) in those cases intensely expressing the molecule. AGASAC frequently expressed PDGFRβ in a high percentage of neoplastic cells and in tumor stromal compartments with high intensity, supporting the addition of TKIs to AGASAC treatment, especially in tubular tumors. Contrarily, loss of PDGFRβ could potentially render the treatment less effective.

Expression of PDGFRβ in neoplastic stromal compartments (CAFs and vasculature) may further provision the use of TKIs, as these drugs may be effective also on the tumor microenvironment [39]. Despite the finding of PDGFRβ in normal glandular epithelium, small-molecule TKIs such as toceranib achieve therapeutic selectivity not merely through target expression intensity. Instead, this selectivity arises because tumor cells and tumor-associated stroma often show constitutive activation of RTK signaling, whereas normal glandular epithelium may express PDGFRβ but remains functionally inactive. In addition, the anti-angiogenic and microenvironmental effects of TKIs mainly disrupt abnormal tumor vasculature rather than the stable vessels of normal tissue. Consequently, higher cell proliferation, leaky vasculature, and greater drug exposure make tumors more susceptible to TKI therapy compared to normal tissue [16,22,40]. Clinical proof of this concept has been demonstrated in dogs with AGASACs, where toceranib treatment has improved outcomes [22], further substantiating our hypothesis.

Our result should be considered alongside published data regarding expression of other tyrosine kinase receptors, including VEGFR [16] and PDGFRα, further supporting adjunctive TKI therapy.

This study poses some limitations. First, its retrospective nature and relatively limited sample size—particularly for the less common histological variants—constrain the statistical robustness and generalizability of the findings. Second, PDGFRβ expression was assessed exclusively by immunohistochemistry, without parallel evaluation of receptor activation status (e.g., phosphorylation) or downstream signaling pathways, which would provide a more comprehensive understanding of its functional relevance as a therapeutic target. In addition, the absence of clinical follow-up and treatment outcome data precluded correlation of PDGFRβ expression with therapeutic response or prognosis. Future prospective studies integrating molecular analyses with clinical outcome data are warranted to further elucidate the predictive and therapeutic significance of PDGFRβ expression in AGASACs.

## 5. Conclusions

To the best of the authors’ knowledge, this is the first study to describe comedo-carcinoma-like and neuroendocrine-like AGASAC subtypes and to assess PDGFRβ expression across different tumor subtypes and their corresponding lymph node metastases. Our findings indicate that PDGFRβ expression varies among tumor types, possibly reflecting differences in tumor differentiation, and that distinguishing these subtypes may aid in AGASAC diagnosis and in selecting adjunctive treatments. The clinical significance of these observations, including their relationship with tumor behavior and prognosis, warrants further investigation.

## Figures and Tables

**Table 1:** Signalment and clinical data of dogs.

**Sex**	
Female	40
Of which neutered	34
Male	11
Of which neutered	4
F/M ratio	3.6
**Age (Years)**	
Mean	10.54
Range	5–15
**Breeds**	
Pure breeds	29
Crossbreeds	22
**Involved anal sac**	
Right	28
Left	20
Unknown	3
**Tumor size (cm)**	
Mean	4.07
Range	0.5–11
**Regional enlarged lymph node**	
Yes	39
No	12
**Lesions compatible with distant metastasis**	
Yes	11
No	40

**Table 2:** Histological evaluation of canine AGASAC and corresponding lymph node metastases.

Tumor Histotype	N. of Cases	Necrosis	Cases with Metastasis	Available Lymph Node Metastasis	Lymph Node Metastasis Histotype ^1^
Mixed with solid prevalence	26	8	19 (73%)	16	17
Pure solid	10	6	8 (80%)	6	8
Solid subtype comedo-carcinoma	1	1	1 (100%)	1	1
Mixed with tubular prevalence	9	0	9 (100%)	9	7
Pure tubular	4	1	1 (25%)	1	0
Neuroendocrine-like (rosettes and packets)	1	1	1 (100%)	0	0
**Total**	51	17	39	33	33

Notes: ^1^, in a subset of tumors, metastasis showed an increased solid pattern compared with the primary tumor.

**Table 3:** Immunohistochemical expression and staining intensity of PDGFRβ in 51 canine primary AGASACs.

	Number of Positive Cells					Staining Intensity		
Tumor Histotype	-	1+	2+	3+	4+	Weak	Moderate	Strong
Mixed with solid prevalence	7	2	8	1	8	3	9	7
Pure solid	1	2	2	3	2	3	3	3
Solid subtype comedo-carcinoma	0	0	0	0	1	0	1	0
Mixed with tubular prevalence	0	1	2	4	2	0	5	4
Pure tubular	0	0	1	1	2	1	3	0
Neuroendocrine-like (rosettes and packets)	0	0	0	1	0	0	1	0
**Total**	8	5	13	10	15	7	22	14

Symbols: -, <5% positive neoplastic cells; 1+, 6–<25% positive neoplastic cells; 2+, 25–<50% positive neoplastic cells; 3+, 50–<75% positive neoplastic cells; 4+, ≥75% positive neoplastic cells.

**Table 4:** Immunohistochemical expression and staining intensity of PDGFRβ in 33 canine AGASAC in lymph node metastases.

		Number of Positive Cells					Staining Intensity		
Lymph Node Metastasis Histotype	N. of Cases	-	1+	2+	3+	4+	Weak	Moderate	Strong
Mixed with solid prevalence	17	5	2	3	3	4	2	6	4
Pure solid	8	0	3	3	0	2	5	2	1
Solid subtype comedo-carcinoma	1	0	1	0	0	0	1	0	0
Mixed with tubular prevalence	7	1	1	2	2	1	2	3	1
Pure tubular	0	0	0	0	0	0	0	0	0
Neuroendocrine-like (rosettes and packets)	0	0	0	0	0	0	0	0	0
**Total**	33	6	7	8	5	7	10	11	6

Symbols: -, <5% positive neoplastic cells; 1+, 6–<25% positive neoplastic cells; 2+, 25–<50% positive neoplastic cells; 3+, 50–<75% positive neoplastic cells; 4+, ≥75% positive neoplastic cells.

**Table 5:** Immunohistochemical expression and staining intensity of PDGFRβ in the tumor microenvironment of primary canine AGASACs.

		Cancer Associated Fibroblasts (CAFs)			Blood Vessels		
Tumor Histotype	N. of Cases	Negative (<5%)	Low Positive (6–50%)	Strong Positive (>51%)	Negative (<5%)	Low Positive (6–50%)	Strong Positive (>51%)
Mixed with solid prevalence	26	2	6	18	2	2	22
Pure solid	10	1	0	9	1	0	9
Solid subtype comedo-carcinoma	1	0	0	1	0	0	1
Mixed with tubular prevalence	9	0	1	8	0	0	9
Pure tubular	4	0	0	4	0	0	4
Neuroendocrine-like (rosettes and packets)	1	0	0	1	0	0	1
**Total**	51	3	7	41	3	2	46

Notes: Stroma positivity was not included in the table as it was consistently negative.

**Table 6:** Immunohistochemical expression and staining intensity of PDGFRβ in the tumor microenvironment of lymph node metastases.

		Cancer Associated Fibroblasts (CAFs)			Blood Vessels		
Lymph Node Metastases Histotype	N. of Cases	Negative (<5%)	Low Positive (6–50%)	Strong Positive (>51%)	Negative (<5%)	Low Positive (6–50%)	Strong Positive (>51%)
Mixed with solid prevalence	17	2	2	13	0	3	14
Pure solid	8	0	0	8	0	0	8
Solid subtype comedo-carcinoma	1	0	0	1	0	0	1
Mixed with tubular prevalence	7	0	1	6	0	1	6
Pure tubular	0	0	0	0	0	0	0
Neuroendocrine-like (rosettes and packets)	0	0	0	0	0	0	0
**Total**	33	2	3	28	0	4	29

Notes: Stroma positivity was not included in the table as it was consistently negative.

**Table 7:** Multiclass analysis and descriptive statistics of positivity and intensity of PDGFRβ scores in canine AGASAC histotypes.

		PDGFRβ Positivity Score							PDGFRβ Intensity Score	
Tumor Histotype	N. of Cases	-	1+	2+	3+	4+	Median	Mean (SD)	Median	Mean (SD)
Mixed with solid prevalence	26	7 (26.92%)	2 (7.69%)	8 (30.77%)	1 (3.85%)	8 (30.77%)	2.00	2.04 (1.59)	2.00	1.65 (1.20)
Pure solid	10	1 (10.00%)	2 (20.00%)	2 (20.00%)	3 (30.00%)	2 (20.00%)	2.5	2.3 (1.34)	2.00	1.8 (1.03)
Solid subtype comedo-carcinoma	1	0 (0.00%)	0 (0.00%)	0 (0.00%)	0 (0.00%)	1 (100.00%)	4.00	4.00	2.00	2.00
Mixed with tubular prevalence	9	0 (0.00%)	1 (11.11%)	2 (22.22%)	4 (44.44%)	2 (22.22%)	3.00	2.78 (0.97)	2.00	2.44 (0.53)
Pure tubular	4	0 (0.00%)	0 (0.00%)	1 (25.00%)	1 (25.00%)	2 (50.00%)	3.50	3.25 (0.96)	2.00	1.75 (0.50)
Neuroendocrine-like (rosettes and packets)	1	0 (0.00%)	0 (0.00%)	0 (0.00%)	1 (100.00%)	0 (0.00%)	3.00	3.00	3.00	3.00
**Total**	51	8	5	13	10	15	-			

PDGFRβ positivity score: Symbols: -, <5% positive neoplastic cells; 1+, 6–<25% positive neoplastic cells; 2+, 25–<50% positive neoplastic cells; 3+, 50–<75% positive neoplastic cells; 4+, ≥75% positive neoplastic cells.

**Table 8:** Summary of Mann–Whitney U test results comparing positivity scores across grouped tumor histotypes.

Grouping	N (Target Group)	Mann–Whitney U	*p*-Value	Mean Rank (Target Group)	Mean Rank (Other Histotypes)	*r* (Effect Size)
Pure tubular and mixed with tubular prevalence	13	180.50	0.142	31.12	24.25	−0.269
Mixed with solid prevalence and pure solid	37	331.00	0.121	24.05	31.14	0.278

## Data Availability

The original contributions presented in this study are included in the article/Supplementary Material. Further inquiries can be directed to the corresponding author(s).

## Supplementary Materials

The following supporting information can be downloaded at: https://www.mdpi.com/article/10.3390/vetsci12121122/s1, Supplemental Table S1. Signalment and clinical data of the dogs included in this study. Supplemental Table S2. Primary tumor and metastasis histotype. Supplemental Material S3. Alignment of canine and human PDGFRβ protein.

## Abbreviations

The following abbreviations are used in this manuscript:

ABC	avidin–biotin complex
AGASAC	apocrine gland anal sac adenocarcinoma
CAF	cancer-associated fibroblast(s)
CT	computed tomography
DAB	3,3’-diaminobenzidine
DIVAS	Department of Veterinary Medicine and Animal Sciences
DVM	Doctor of Veterinary Medicine
ECVP	European College of Veterinary Pathologists
EMT	epithelial–mesenchymal transition
FFPE	formalin-fixed paraffin-embedded
H&E	hematoxylin and eosin
KIT	mast/stem cell growth factor receptor (CD117)
OPBA	Organo Preposto al Benessere Animale (Institutional Animal Welfare Body)
PDGF	platelet-derived growth factor
PDGFR	platelet-derived growth factor receptor
PDGFRβ	platelet-derived growth factor receptor beta
RNA	ribonucleic acid
RTK	receptor tyrosine kinase(s)
TKI	tyrosine kinase inhibitor(s)
VEGF	vascular endothelial growth factor
VEGFR2	vascular endothelial growth factor receptor 2
