# Supplementary material for: PDGFRβ Expression Across Canine AGASAC Subtypes and Metastases: Morphologic Insights and Possible Therapeutic Implications
Source: Vet Sci. 2025 Nov 26;12(12):1122. doi: 10.3390/vetsci12121122 (PMC12737537; doi:10.3390/vetsci12121122)
Supplement: Supplementary file 1 [file vetsci-12-01122-s001.zip › Supplemental Material S3.pdf]

Supplemental Material S3. Alignment of canine and human PDGFRβ protein.

```
CLUSTAL 2.1 Multiple Sequence Alignments


Sequence type explicitly set to Protein
Sequence format is Pearson
Sequence 1: sp|P09619|PDGFRB_human   1106 aa
Sequence 2: sp|Q6QNF3|PDGFRB_canine  1103 aa
Start of Pairwise alignments
Aligning...



Sequences (1:2) Aligned. Score: 89.7552
Guide tree file created:   [clustalw.dnd]

There are 1 groups
Start of Multiple Alignment

Aligning...
Group 1: Sequences:   2      Score:17165
Alignment Score 6227

sp|P09619|PDGFRB_human       MRLPGAMPALALKGELLLLSLLLLLEPQISQGLVVTPPGPELVLNVSSTF
sp|Q6QNF3|PDGFRB_canine      MQVPGTMPAPVLKGQALWLPLLLMLSPQASGGLVITPPGPELVLNISSTF
                             *::**:*** .***: * *.***:*.** * ***:**********:****

sp|P09619|PDGFRB_human       VLTCSGSAPVVWERMSQEPPQEMAKAQDGTFSSVLTLTNLTGLDTGEYFC
sp|Q6QNF3|PDGFRB_canine      VLTCSGPAPVVWERLSQEPLQKMARTQDGTFSSTLTLTNVTGLDTGEYFC
                             ******.*******:**** *:**::*******.*****:**********

sp|P09619|PDGFRB_human       THNDSRGLETDERKRLYIFVPDPTVGFLPNDAEELFIFLTEITEITIPCR
sp|Q6QNF3|PDGFRB_canine      TYKGSHGLEPDGRKRLYIFVPDPTMGFLPVDPEELFIFLTEITEITIPCR
                             *::.*:***.* ************:**** *.******************

sp|P09619|PDGFRB_human       VTDPQLVVTLHEKKGDVALPVPYDHQRGFSGIFEDRSYICKTTIGDREVD
sp|Q6QNF3|PDGFRB_canine      VTDPRLVVTLHEKKVDIPLPIPYDHQRGFSGTFEDKTYVCKTTIGDKEVD
                             ****:********* *:.**:********** ***::*:*******:***

sp|P09619|PDGFRB_human       SDAYYVYRLQVSSINVSVNAVQTVVRQGENITLMCIVIGNEVVNFEWTYP
sp|Q6QNF3|PDGFRB_canine      SEAYYVYSLQVSSINVSVNAVQTVVRQGENITIMCIVTGNEVVNFEWTYP
                             *:***** ************************:**** ************

sp|P09619|PDGFRB_human       RKESGRLVEPVTDFLLDMPYHIRSILHIPSAELEDSGTYTCNVTESVNDH
sp|Q6QNF3|PDGFRB_canine      RMESGRLVEPVTDFLFNVPSHIRSILHIPSAELGDSGTYICNVSESVNDH
                             * *************:::* ************* ***** ***:******

sp|P09619|PDGFRB_human       QDEKAINITVVESGYVRLLGEVGTLQFAELHRSRTLQVVFEAYPPPTVLW
sp|Q6QNF3|PDGFRB_canine      RDEKSINVTVVESGYVRLLGELDAVQFAELHRSRALQVVFEAYPPPTVVW
                             :***:**:*************:.::*********:*************:*

sp|P09619|PDGFRB_human       FKDNRTLGDSSAGEIALSTRNVSETRYVSELTLVRVKVAEAGHYTMRAFH
sp|Q6QNF3|PDGFRB_canine      FKDNRTLGDSSAGEIALSTRNVSETRYVSELTLVRVKVAEAGYYTMRAFH
                             ******************************************:*******

sp|P09619|PDGFRB_human       EDAEVQLSFQLQINVPVRVLELSESHPDSGEQTVRCRGRGMPQPNIIWSA
sp|Q6QNF3|PDGFRB_canine      EDAEAQLSFQLQVNVPVRVLELSESHPASGEQTVRCRGRGMPQPHLTWST
                             ****.*******:************** ****************:: **:

sp|P09619|PDGFRB_human       CRDLKRCPRELPPTLLGNSSEEESQLETNVTYWEEEQEFEVVSTLRLQHV
sp|Q6QNF3|PDGFRB_canine      CSDLKRCPRELPPTPLGNSSEEESQLETNVTYWPEDQEFEVVSTLRLRRV
                             * ************ ****************** *:***********::*

sp|P09619|PDGFRB_human       DRPLSVRCTLRNAVGQDTQEVIVVPHSLPFKVVVISAILALVVLTIISLI
sp|Q6QNF3|PDGFRB_canine      DQPLSVRCTLHNLLGHDMQEVTVVPHSLPFKVVVISAILALVVLTIISLI
                             *:********:* :*:* *** ****************************
```

```
sp|P09619|PDGFRB_human       ILIMLWQKKPRYEIRWKVIESVSSDGHEYIYVDPMQLPYDSTWELPRDQL
sp|Q6QNF3|PDGFRB_canine      ILIMLWQKKPRYEIRWKVIESVSSDGHEYIYVDPMQLPYDSTWELPRDQL
                             **************************************************

sp|P09619|PDGFRB_human       VLGRTLGSGAFGQVVEATAHGLSHSQATMKVAVKMLKSTARSSEKQALMS
sp|Q6QNF3|PDGFRB_canine      VLGRTLGSGAFGQVVEATAHGLSHSQATMKVAVKMLKSTARSSEKQALMS
                             **************************************************

sp|P09619|PDGFRB_human       ELKIMSHLGPHLNVVNLLGACTKGGPIYIITEYCRYGDLVDYLHRNKHTF
sp|Q6QNF3|PDGFRB_canine      ELKIMSHLGPHLNVVNLLGACTKGGPIYIITEYCRYGDLVDYLHRNKHTF
                             **************************************************

sp|P09619|PDGFRB_human       LQHHSDKRRPPSAELYSNALPVGLPLPSHVSLTGESDGGYMDMSKDESVD
sp|Q6QNF3|PDGFRB_canine      LQLCSDKRRPPSAELYSNALPAGLPLPSHVSLPGESDGGYMDMSKDESVD
                             **  *****************.**********.*****************

sp|P09619|PDGFRB_human       YVPMLDMKGDVKYADIESSNYMAPYDNYVPSAPERTCRATLINESPVLSY
sp|Q6QNF3|PDGFRB_canine      YVPMLDMKGGVKYADIESSSYMAPYDNYVPTAPERTCRATLINESPVLSY
                             *********.*********.**********:*******************

sp|P09619|PDGFRB_human       MDLVGFSYQVANGMEFLASKNCVHRDLAARNVLICEGKLVKICDFGLARD
sp|Q6QNF3|PDGFRB_canine      TDLVGFSYQVANGMEFLASKNCVHRDLAARNVLICEGKLVKICDFGLARD
                              *************************************************

sp|P09619|PDGFRB_human       IMRDSNYISKGSTFLPLKWMAPESIFNSLYTTLSDVWSFGILLWEIFTLG
sp|Q6QNF3|PDGFRB_canine      IMRDSNYISKGSTFLPLKWMAPESIFNSLYTTLSDVWSFGILLWEIFTLG
                             **************************************************

sp|P09619|PDGFRB_human       GTPYPELPMNEQFYNAIKRGYRMAQPAHASDEIYEIMQKCWEEKFEIRPP
sp|Q6QNF3|PDGFRB_canine      GTPYPELPMNEQFYNAIKRGYRMAQPAHASDEIYEIMQKCWEEKFEIRPP
                             **************************************************

sp|P09619|PDGFRB_human       FSQLVLLLERLLGEGYKKKYQQVDEEFLRSDHPAILRSQARLPGFHGLRS
sp|Q6QNF3|PDGFRB_canine      FSQLVLLLERLLGEGYKKKYQQVDEEFLRSDHPAVLRSQARLPGFPGLRS
                             **********************************:********** ****

sp|P09619|PDGFRB_human       PLDTSSVLYTAVQPNEGDNDYIIPLPDPKPEVADEGPLEGSPSLASSTLN
sp|Q6QNF3|PDGFRB_canine      PLDTSSVLYTAVQPNEGDNDYIIPLPDPKPEVADG-PLESSPSLASSTLN
                             **********************************  ***.**********

sp|P09619|PDGFRB_human       EVNTSSTISCDSPLEPQDEPEPEPQLELQVEPEPELEQLPDSGCPAPRAE
sp|Q6QNF3|PDGFRB_canine      EVNTSSTISCDSPLEPQEEPEPEP--EPQPEPQVVPEPPLDSSCPGPRAE
                             *****************:******  * * **:   *   **.**.****

sp|P09619|PDGFRB_human       AEDSFL
sp|Q6QNF3|PDGFRB_canine      AEDSFL
                             ******
```
