# Supplementary material for: PDGFRβ Expression Across Canine AGASAC Subtypes and Metastases: Morphologic Insights and Possible Therapeutic Implications
Source: Vet Sci. 2025 Nov 26;12(12):1122. doi: 10.3390/vetsci12121122 (PMC12737537; doi:10.3390/vetsci12121122)
Supplement: Supplementary file 1 [file vetsci-12-01122-s001.zip › Supplemental Table S1.pdf]

Supplemental Table S1. Signalment and clinical data of the dogs included in the study

Dog	Breed	Sex	Age (years)	Hypercalcemia	Tumour dimension (cm)	Localization	Regional Metastasis	Distant metastasis
1	Boxer	F	9	No	2,5	Left	Yes	No
2	German shepherd	F	9	Yes	5	Left	Yes	No
3	Mixed Breed	FS	12	No	6	Left	Yes	Yes
4	Mixed Breed	F	15	Yes	6	Right	Yes	Yes
5	Labrador retriever	MC	12	No	3	Right	Yes	No
6	Mixed Breed	FS	12	No	1,5	Left	Yes	Yes
7	Mixed Breed	FS	10	No	0,5	Left	Yes	No
8	Mixed Breed	FS	8	No	3	Right	No	No
9	Border Collie	FS	11	No	5	Left	Yes	No
10	Labrador retriever	FS	11	Yes	9	Right	No	No
11	Mixed Breed	FS	9	Yes	1,5	NA	Yes	No
12	Mixed Breed	FS	14	No	2,5	NA	No	No
13	Cavalier King Charles spaniel	FS	10	No	3	Right	Yes	Yes
14	Mixed Breed	FS	9	No	10	Right	Yes	Yes
15	Mixed Breed	FS	7	No	4	Right	Yes	Yes
16	German shepherd	FS	11	No	3	Right	Yes	Yes
17	Siberian husky	FS	13	No	6,5	Left	No	No
18	Beagle	FS	10	Yes	3	Right	Yes	No
19	Border Collie	FS	9,5	Yes	2	Right	Yes	Yes

20	Mixed Breed	M	12	No	5	NA	Yes	Yes
21	Mixed Breed	MC	12	Yes	5	Right	No	No
22	Mixed Breed	FS	13	No	7	Right	Yes	Yes
23	Labrador retriever	M	11	Yes	1	Left	Yes	No
24	Border collie	M	10	No	4	Right	No	No
25	Basset hound	FS	7	Yes	2	Right	Yes	No
26	Mixed Breed	FS	11	No	4	Left	No	No
27	Mixed Breed	FS	14	No	3	Right	No	No
28	Labrador retriever	MC	12,5	No	2	Left	Yes	No
29	Labrador retriever	FS	10	No	1,5	Left	No	No
30	Labrador retriever	MC	11	No	6	Right	No	No
31	Mixed Breed	FS	9,5	Yes	5	Right	Yes	Yes
32	Labrador retriever	F	13	Yes	3	Left	Yes	No
33	Mixed Breed	FS	9	Yes	2,2	Left	Yes	No
34	English setter	FS	12	No	3	Right	Yes	No
35	Czechoslovakian Wolfdog	F	11	No	5	Right	Yes	No
36	Czechoslovakian Wolfdog	FS	7	Yes	3,5	Left	Yes	No
37	Mixed Breed	M	8	No	6	Right	Yes	No
38	Labrador retriever	FS	10	No	7,4	Left	Yes	No
39	Labrador retriever	FS	12,5	NA	5	Right	Yes	No
40	Dachshund	M	13,5	NA	7	Left	Yes	No

41	Mixed Breed	FS	11	NA	3,5	Left	Yes	No
42	Golden retriever	FS	8	Yes	0,7	Left	Yes	No
43	Mixed Breed	FS	11,5	NA	5,5	Right	Yes	Yes
44	Cocker spaniel	F	10,5	NA	4	Left	Yes	No
45	Jack Russel terrier	M	10	NA	5	Left	Yes	No
46	Border Collie	M	10	NA	2	Right	Yes	No
47	Hungarian Vizsla	FS	11	NA	4	Right	Yes	No
48	Mixed Breed	FS	15	NA	11	Right	No	No
49	Mixed Breed	FS	10	NA	3	Right	Yes	No
50	Mixed Breed	FS	10	NA	4	Right	No	No
51	Cavalier King Charles Spaniel	FS	7	NA	0,7	Right	Yes	No

F, female; FS, spayed female; M, male; MC, castrated male; NA, not available
