# Supplementary material for: PDGFRβ Expression Across Canine AGASAC Subtypes and Metastases: Morphologic Insights and Possible Therapeutic Implications
Source: Vet Sci. 2025 Nov 26;12(12):1122. doi: 10.3390/vetsci12121122 (PMC12737537; doi:10.3390/vetsci12121122)
Supplement: Supplementary file 1 [file vetsci-12-01122-s001.zip › Supplemental Table S2.pdf]

Supplemental Table S2. Primary tumor and metastasis histotype.

Dog	Tumor histotype	Metastases histotype
1	Tubular	MTP
2	MSP	MSP
3	Solid	NA
4	Solid	Solid
5	MTP	MTP
6	MSP	MSP
7	MTP	MTP
8	MSP	Not metastatic
9	Comedo-carcinoma	Comedo-carcinoma
10	Tubular	Not metastatic
11	Solid	Solid
12	Solid	Not metastatic
13	MTP	MSP
14	MSP	MSP
15	Solid	Solid
16	MSP	MSP
17	Solid	Not metastatic
18	MTP	MTP
19	MSP	MSP
20	Neuroendocrine packets	NA
21	MSP	Not metastatic
22	MSP	Not metastatic
23	MTP	MSP
24	Tubular	Not metastatic
25	MTP	MSP
26	MSP	Not metastatic
27	MSP	Not metastatic
28	MSP	MSP
29	MSP	Not metastatic
30	Tubular	Not metastatic
31	Solid	Solid
32	MTP	MTP
33	MSP	Solid
34	MSP	MSP
35	Solid	Solid
36	MSP	Solid
37	MTP	MTP
38	Solid	Solid
39	MSP	MSP

40	MSP	MSP
41	MSP	MSP
42	MTP	MTP
43	MSP	MSP
44	Solid	NA
45	MSP	MSP
46	MSP	NA
47	MSP	NA
48	MSP	NA
49	MSP	MSP
50	MSP	Not metastatic
51	MSP	MSP

Symbols: MSP, mixed with solid prevalence; MTP, mixed with tubular prevalence; NA, metastasis sample not available
